# Pain Assessment of Horses With Trigeminal‐Mediated Headshaking (TMHS) at Rest Between Episodes

**DOI:** 10.1111/jvim.70064

**Published:** 2025-04-01

**Authors:** Vanessa Franzen, Daniela Reisbeck, Yvonne Leibl, Angelika Schoster, Anna May

**Affiliations:** ^1^ Equine Hospital Ludwig Maximilians University Munich Munich Germany

**Keywords:** equine, headshaking, neuropathic pain, pain scale, trigeminus

## Abstract

**Background:**

While there is agreement that horses experience pain during the actual headshaking attacks, there is still a lack of research about the time between the individual episodes in this species.

**Objectives:**

To evaluate the signs of pain in horses with TMHS at rest in the absence of common triggering factors.

**Animals:**

Twenty TMHS horses (not head shaking at the time of pain assessment), 20 horses with colic, and 20 clinically healthy horses.

**Methods:**

Descriptive study assessing pain in TMHS horses during the absence of clinical signs using the Horse Grimace scale (HGS). To grade the pain shown with TMHS, horses with moderate gastrointestinal pain conditions and a healthy horses' group were also included. For evaluation, videos were taken on two consecutive days from both sides of the face (healthy, TMHS) or before analgesics were applied (colic). Eight photos per horse were extracted from the videos, randomized, and evaluated by three examiner groups, each comprising two persons: (a) last‐year veterinary medicine students without previous use of the HGS, (b) experienced veterinarians without experience in using the HGS, and (c) experienced veterinarians who regularly apply the HGS. All examiners were blinded to the underlying condition.

**Results:**

Median HGS scores in the groups (healthy, gastrointestinal, TMHS) differed significantly from each other (*p* < 0.05). Healthy horses had median HGS scores below 2 (median 1.2, range: 0.1–2.4), whereas TMHS horses had higher median HGS scores (5.6, range 2.4–7.4), and colic horses had the highest HGS scores (6.6, range: 3.8–8.4). There was a statistically significant difference for all facial action units (FAU) when the different horse groups were compared (*p* < 0.05). Effect sizes were large for the healthy horses' group (ES‐1.23) and for the colic horses (ES 0.86), and small to medium for the TMHS horses (ES 0.37).

**Clinical Importance:**

The results of this study show that pain occurs between individual TMHS attacks in severely affected horses and that the quality of life of these horses must be questioned and evaluated individually.

**Abbreviations**
FAU: facial action unit
FLASH: fast localized abdominal sonography of horses
HGS: Horse Grimace scale
TMHS: trigeminal‐mediated headshaking
TN: trigeminal neuralgia

## 1 Introduction

Trigeminal‐mediated headshaking (TMHS) in horses is caused by neuropathic pain and has no obvious underlying cause [1, 2, 3]. The trigeminal nerve becomes sensitive and has a reduced stimulus threshold, so that even mild stimuli such as light, wind, touch, or noise trigger severe pain in affected horses [4, 5]. The pain associated with this condition in horses is comparable to trigeminal neuralgia (TN) in humans and is characterized by electric shock‐like severe pain that impairs the horse's welfare [6, 7, 8]. Affected horses show violent flicking movements of the head and neck as well as other clinical signs such as snorting, nose rubbing, and increased lip or tongue movements [9, 10].

The actual attacks are tormenting for affected horses, but painful sensations in between the episodes could be possible and should be investigated to evaluate the horses' quality of life.

Pain is difficult to objectify, especially in veterinary medicine [11]. Since the methods of questioning and self‐reporting cannot be used in animals to capture the emotional component of pain, behavioral changes must be used primarily [12, 13]. Chronic neuropathic pain is a complex phenomenon, and a major problem is the recognition of this specific pain, especially in horses that do not enunciate pain with sounds and obvious expressions like other species. It is therefore usually only accompanied by behavioral changes, and many veterinarians are not sufficiently trained in recognition, as there is no universal pain scale suitable for evaluation [14, 15, 16].

The Horse Grimace scale (HGS) is a tool to assess pain in horses based on facial expression and six so‐called facial action units (FAU) [12]. It has already been used and confirmed in many studies, for example, to categorize pain during elective surgery (castrations) or in horses with acute laminitis, pain induced by clamping or after the application of capsaicin [12, 13, 17, 18, 19]. In studies, the HGS was not distorted by emotional states without pain and therefore serves specifically for pain detection [18].

The aim of this study was to evaluate pain between headshaking episodes in horses diagnosed with TMHS and to gain more insight on the welfare impairment of affected horses. Moreover, the usability of the HGS in terms of comparability between differently experienced observers and the reliable detection of pain in headshaking horses (by comparison with painful colic states) was evaluated in this study. Furthermore, the study aimed to understand the neuropathic pain face of a species that does not express obvious signs and sounds of pain.

## 2 Materials and Methods

### 2.1 Study Design

This descriptive study was performed between May 2022 and May 2024, where recruitment of TMHS horses took place.

### 2.2 Animals

This study comprised 20 horses with TMHSTMHS, 20 horses with moderate gastrointestinal disease, and 20 healthy horses. The TMHS group eligible population comprised members of the source population that were presented for examination and treatment at the equine hospital of Ludwig‐Maximilians‐University between May 2022 and May 2024. As a comparison for the grade of pain, horses with acute moderate gastrointestinal diseases were filmed between February 2021 and May 2023 in the home stable environment. The healthy comparison group study population comprised 20 horses from a single privately owned boarding stable that were not presented for medical issues or medical treatment within the past 6 months. All horses were client‐owned, and owners consented to the use of photos.

#### 2.2.1 TMHS Group (T)

Horses diagnosed with severe TMHS by two senior clinicians (board‐eligible and board‐certified) in the equine clinic of LMU Munich between May 2022 and May 2024 were eligible for inclusion. Severe headshaking was defined as frequent headshaking signs displayed every day to an extent that horses could not be ridden or used otherwise. Accordingly, inclusion criteria were headshaking attacks clearly displayed by the horses during the time and on the day the videos were taken and a duration of clinical signs for more than 1 year. Headshaking due to reasons other than primary trigeminal‐mediated pain was ruled out according to a fixed protocol, and horses were only included if all examinations were without clinically significant findings. The examination protocol consisted of a general examination, exercise test, neurologic examination, ophthalmic examination in mydriasis (direct and indirect ophthalmoscopy, slit lamp), examination of the oral cavity/teeth using a mouth speculum and a teeth endoscope under sedation, ear examination and otoscopy, endoscopy of upper and lower airways, including guttural pouches, computed tomography of the head, lateral radiographs of the neck (caudal occiput to first thoracic vertebra), and blood examination (complete blood count and serum parameters, serum amyloid A). Exclusion criteria were other medical issues (e.g., lameness) or medical treatment within the past 6 months.

#### 2.2.2 Gastrointestinal Diseases/Colic Group (C)

The videos of the horses with gastrointestinal diseases were taken between February 2021 and May 2023 by the referring veterinarians in the horses' home stable environment according to the study protocol (see section 2.5) before 8 out of 20 were sent to the hospital for further diagnostics. All cases were nonsurgical cases that resolved with conservative treatment.

All horses showed mild to moderate colic signs (inappetence, pawing, flehmen, sternal, and lateral recumbency) and had heart rates ranging from 48and 60 per minute, respiratory rates between 16 and 28 per minute, and normal rectal temperatures. The cardiovascular system of the horses included in this study was not severely affected, and they had pink mucous membranes and capillary refill times below 2 s. Horses were filmed before the administration of analgesics, and videos of suitable horses were included after a diagnosis had been made by the veterinarian with further diagnostics: blood work, nasogastric intubation, abdominal FLASH ultrasound, transrectal examination, and abdominocentesis. Horses with the following gastrointestinal conditions were included: severe colonic impactions (7/20), nephrosplenic entrapment (5/20), tympanism (5/20), and colonic displacements (3/20).

#### 2.2.3 Healthy Horses' Group (H)

Horses without any medical condition based on a normal general examination were included in this group of horses. Horses were all recruited from one boarding stable and filmed in the home stable environment by two clinicians of the equine hospital who were not involved in the grading afterwards. Exclusion criteria were any painful issues or medical treatment within the past 6 months.

### 2.3 Pain Assessment in Horses Using the HGS [12]

The HGS uses six facial regions of the horse, so‐called FAU. The units comprise all regions of the equine face: ears (FAU no. 1, stiffly backwards ears), eyes (FAU no. 2, orbital tightening, FAU no. 3, tension above the eyes), masseter (FAU no. 4, prominent strained chewing muscles), and mouth (FAU no. 5, mouth strained and pronounced chin, FAU no. 6, strained nostrils and flattening of the profile). Each FAU is given a score from 0 (not present), 1 (moderately present), or 2 (obviously present) [12]. The HGS has been assessed in various settings and has been shown to have excellent interobserver reliability [20].

### 2.4 Evaluation Conduct

To investigate whether the answers differed with variable levels of experience of the observers, three groups of observers were chosen. The first group (nonexperienced vets, no experience with HGS—non‐expHGS‐) consisted of two students in the last year of veterinary science studies who had never used the HGS before and were instructed for about 30 min before evaluations, the second group (experienced vets, no experience with HGS—ExpHGS‐) were junior clinicians (2 years' experience in equine practice) with no experience in using the HGS, while the third group (experienced vets, experienced with HGS—ExpHGS+) consisted of two senior clinicians who had over 10 years of experience and were using the HGS regularly.

### 2.5 Study Protocol

Horses were filmed by clinicians of the clinic (healthy), the owners (TMHS) or referring veterinarians (colic) in the usual environment in the stable. The healthy horses and headshakers were filmed on two different days, and the gastrointestinal horses during the colic episode before the administration of analgesics. Videos were recorded from both sides of the face for 2 min each after at least 10 min of acclimatization or when the horses stopped interacting with the person around. A total of 8 images per horse were extracted from the videos, 4 images per side at one timepoint (colic) or 2 images per side at 2 different timepoints (healthy/TMHS). The 8 images were extracted by a nonparticipating person who was blinded to the underlying condition and timepoint. The person recorded the next moment where the horse presented the head's profile to the camera after every 20 (colic) or 40 (healthy/TMHS) seconds. Pain assessment was performed using the HGS and the associated FAU. Examiners, who scored the horses in the pictures, were blinded to the underlying condition (TMHS/gastrointestinal/healthy).

### 2.6 Cutoff Values for Healthy Horses and Mean Pain Scores for all Groups

Median HGS scores for cutoff values for healthy horses and the painful groups (colic, TMHS) were obtained by taking the combined median score of all groups (non‐expHGS‐, ExpHGS‐, ExpHGS+).

### 2.7 Intraobserver Reliability

One week later, the observers of the different groups (non‐expHGS‐, ExpHGS‐, ExpHGS+) were again shown copies of pictures obtained from 5 horses, which had been evaluated before, and repeated the scoring.

### 2.8 Statistical Analysis

Statistical data analyses were performed using SPSS Version 19.0 statistical software package (IBM SPSS Statistics for Mac, Version 19.0. Armonk, NY: IBM Corp.) with statistical significance set at *p* < 0.05.

Data were not normally distributed, and values are thus expressed as the median with the interquartile range. Nonparametric Kruskal‐Wallis ANOVA was used to compare three groups (healthy, gastrointestinal, and TMHS). HGS scores were summarized using medians and ranges. The Spearman correlation test was used to determine correlations between variables. Intraobserver reliability was tested with the intraclass correlation coefficient (ICC). Effect sizes (ES) were reported to measure the degree of linear relationship between two quantitative variables (individual variables compared to total group) with effect sizes defined as small (± 0.2), medium (± 0.5), and large (± 0.8). Figures were created using Prism 9.0 (GraphPad Software Inc. La Jolla, California, USA).

## 3 Results

### 3.1 Animals in the Three Subgroups (TMHS, Gastrointestinal, Healthy)

The group of healthy horses (H) comprised 11 mares, 8 geldings, and one stallion with a median age of 10.5 (minimum age 4, maximum age 18) years; the gastrointestinal/colic horses (C) were 10 mares, 9 geldings, and 1 stallion with a median age of 11 (minimum age 2, maximum age 24) years, whereas the TMHS group (T) consisted of 7 mares and 13 geldings with a median age of 10 (minimum age 4, maximum age 19) years. Various breeds were included, and different breeds were homogenously presented in all groups: the H group consisted of 2 Icelandic horses, 11 Warmbloods, 1 Friesian, 5 Riding Ponies, and 1 Haflinger; the C group comprised 2 Icelandic Horses, 10 Warmbloods, 1 Friesian, 3 Riding Ponies, 1 Haflinger, 2 Quarter Horses, and 1 Purebred Spanish horse; and group T included 12 Warmbloods, 1 Friesian, 1 Riding Pony, 1 Haflinger, and 5 Quarter Horses.

### 3.2 Pain Assessment (Group H, C, T, Total)

#### 3.2.1 Cut‐Off Values for Nonpainful Horses

The median HGS score for healthy horses in this study was 1.2 (range: 0.1–2.4). As there are no reported cut‐off values for the HGS, the cut‐off value for healthy horses not experiencing pain was set at a total score below 2.5 based on the findings obtained in this study.

#### 3.2.2 Median Total Scores (H, C, T)

The median HGS scores in the groups (healthy, gastrointestinal, TMHS) differed significantly from each other (*p* < 0.05). Healthy horses (H) had median HGS scores below 2 (median 1.2, range: 0.1–2.4), whereas TMHS (T) horses had higher median HGS scores (5.6, range: 2.4–7.4), and horses suffering from gastrointestinal conditions (C) had the highest HGS scores (6.6, range: 3.8–8.4).

#### 3.2.3 Median Scores of Individual FAU

**FIGURE 1:**
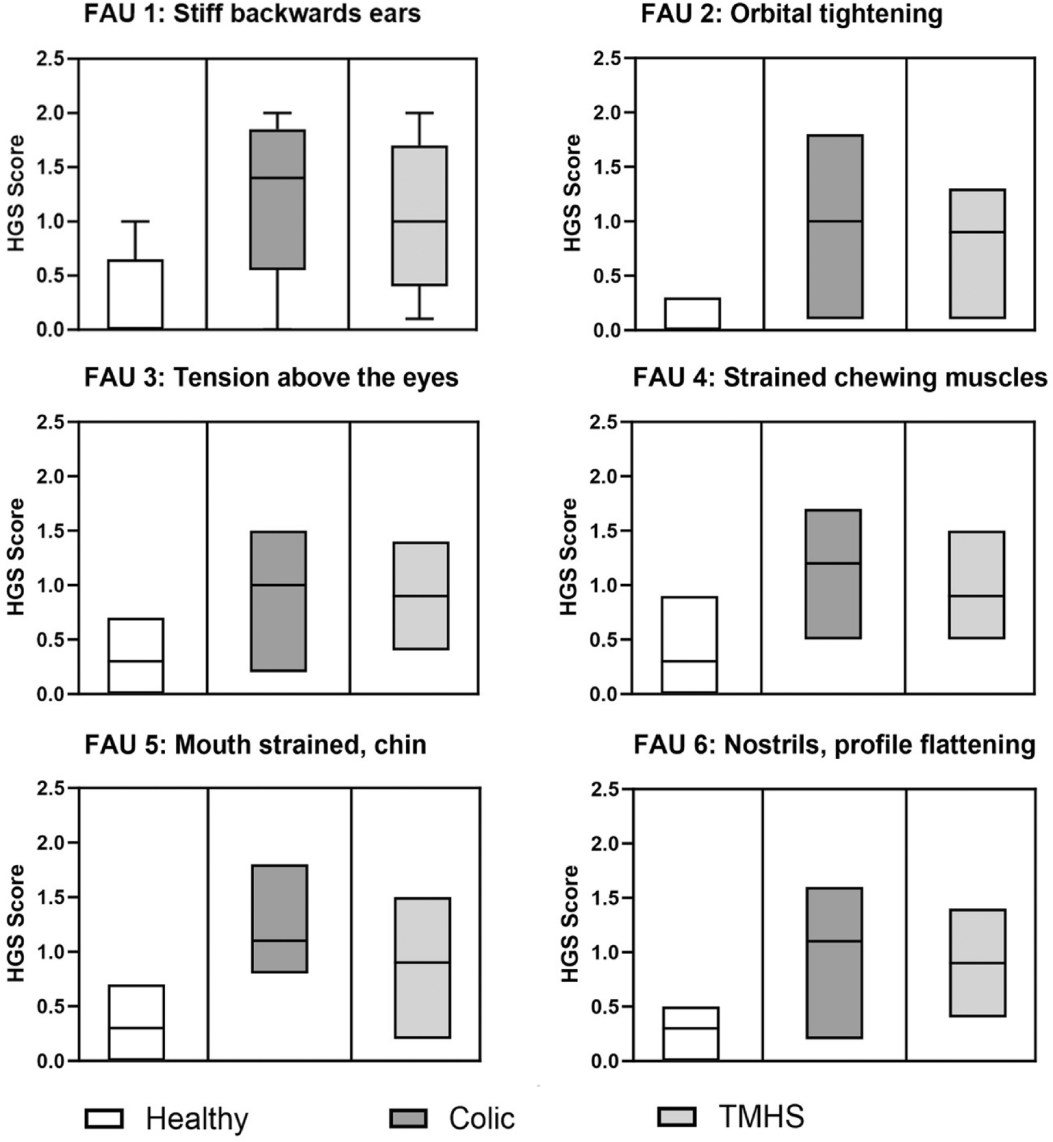
HGS scores (0–2) for individual FAU 1–6 in healthy horses (H), horses with gastrointestinal colic (C), and horses with TMHS (T); median and range shown; *n* = 20 in each group.

There was a statistically significant difference for all FAU when the different horse groups (H, C, T) were compared (*p* < 0.05). Effect sizes (ES) were large for the healthy horses' group (ES ‐1.23) and for the colic horses (ES 0.86), and small to medium for the TMHS horses (ES 0.37; Table 1, Figure 1).

**TABLE 1:** FAU of the individual groups and total FAU scores based on the HGS in three groups of horses (H = healthy, C = colic, T = TMHS; each *n* = 20).

Facial action unit	Horse groups (*n* = 20 each, *n* = 60 total)	Median	Minimum/maximum	ES compared to total group (95% CI)
1 Stiff backwards ears	H	0	0.0, 1.0	−1.03 (0.02–0.3)
	C	1.4	0.0, 2.0	0.77 (1.1–1.6)
	T	1	0.1, 2.0	0.26 (0.8–1.3)
	Total		0.0, 2.0	(0.7–1.0)
2 Orbital tightening	H	0	0.0, 0.3	−1.11 (0.02–0.1)
	C	1	0.1, 1.8	0.65 (0.7–1.1)
	T	0.9	0.1, 1.3	0.46 (0.7–1.0)
	Total		0.0, 1.8	(0.5–0.7)
3 Tension above the eye	H	0.25	0.0, 0.7	−1.07 (0.2–0.4)
	C	1	0.2, 1.5	0.54 (0.8–1.1)
	T	0.9	0.4, 1.4	0.53 (0.8–1.1)
	Total		0.0, 1.5	(0.6–0.8)
4 Strained chewing muscles	H	0.3	0.0, 0.9	−0.99 (0.2–0.5)
	C	1.2	0.5, 1.7	0.78 (1.0–1.3)
	T	0.9	0.5, 1.5	0.21 (0.8–1.0)
	Total		0.0, 1.7	(0.7–0.9)
5 Mouth, pronounced chin	H	0.3	0.0, 0.7	−1.08 (0.2–0.4)
	C	1.1	0.8, 1.8	0.86 (1.1–1.3)
	T	0.85	0.2, 1.5	0.22 (0.8–1.04)
	Total		0.0, 1.8	(0.7–0.9)
6 Strained nostrils, profile	H	0.25	0.0, 0.5	−1.08 (0.2–0.3)
	C	1.1	0.2, 1.6	0.82 (1.0–1.3)
	T	0.85	0.4, 1.4	0.26 (0.7–1.0)
	Total		0.0, 1.6	(0.6–0.9)
Total pain score	H	1.15	0.1, 2.4	−1.23 (1.1–1.8)
	C	6.6	3.8, 8.4	0.86 (6.1–7.2)
	T	5.6	2.4, 7.4	0.37 (4.8–6.1)
	Total		0.1, 8.4	(3.9–5.2)

*Note:* Median values, range, standard error (SE), 95% confidence interval (CI), and effect sizes (ES) compared to the total group (*n* = 60) are shown.

#### 3.2.4 Comparison Between Different Observer Groups/Interobserver Reliability

**FIGURE 2:**
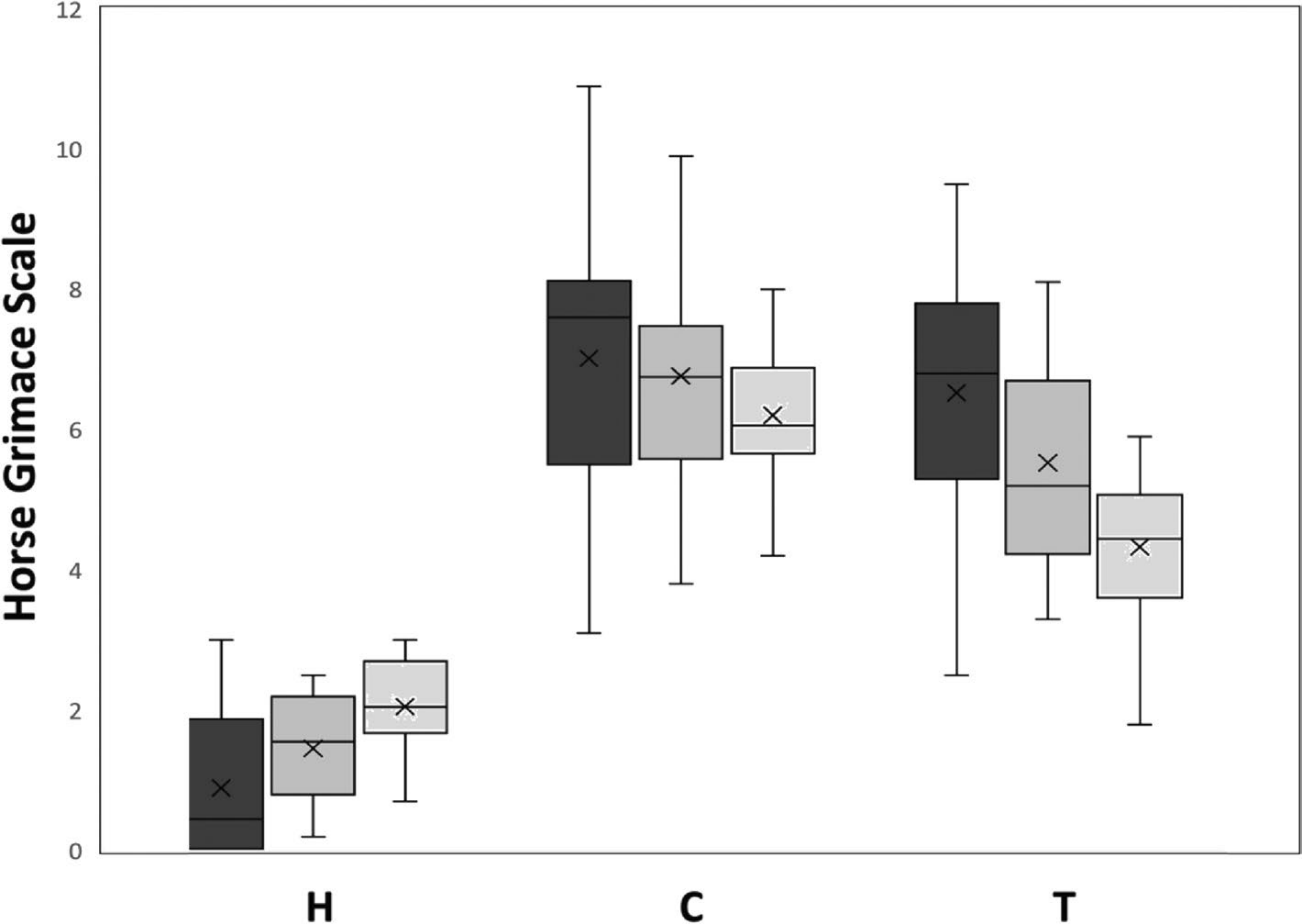
HGS scores evaluated in three groups of horses (H = healthy, C = colic, T = TMHS, *n* = 20 each) by three observer groups (Dark gray: ExpHGS+ = two veterinarians with experience in HGS, medium gray: ExpHGS‐ = two veterinarians without experience in HGS, light gray: Non‐expHGS‐ = two final year veterinary students without experience in HGS). Median and range shown.

There was a statistically significant difference between the scores when the observer groups were compared (*p* < 0.05). Students (non‐expHGS‐) tended to give higher scores in healthy horses, whereas pain associated with TMHS was harder for this observer group to detect. Most experienced examiners (ExpHGS+) gave higher values in the TMHS horses, which were almost comparable to the colic group, while the colic horses were scored similarly by all examiners (Figure 2).

#### 3.2.5 Intraobserver Reliability

The intraobserver reliability between the first and second grading was good to excellent for the individual variables in the observer groups for FAU 1, 2, 3, and 6. For non‐expHGS, the ICC exceeded the variability across groups in FAU 4 and 5, and for ExpHGS in FAU 4 (Table 2).

**TABLE 2:** Intra‐observer reliability of the FAU based on five horses (one healthy, two colic, two TMHS) examined twice by three groups of observers with differing experience.

	FAU 1 (95% CI)	FAU 2 (95% CI)	FAU 3 (95% CI)	FAU 4 (95% CI)	FAU 5 (95% CI)	FAU 6 (95% CI)
ICC non‐expHGS‐	0.93 (−0.09–1.7)	0.96 (0.33–1.1)	0.64 (0.73–1.31)	−0.68 (0.5–1.14)	0.60 (0.69–0.99)	0.70 (0.5–0.98)
ICC ExpHGS—	0.96 (0.42–1.38)	1.00 (0.41–1.19)	0.96 (0.41–1.19)	0.65 (0.7–1.34)	0.97 (0.63–1.04)	0.73 (0.56–1.2)
ICC ExpHGS+	0.95 (0.49–1.67)	0.98 (0.42–1.3)	0.98 (0.6–1.24)	0.98 (0.7–1.22)	0.99 (0.98–1.49)	0.98 (0.43–1.32)
ICC Total	0.95 (0.44–1.52)	0.71 (0.36–1.72)	0.90 (0.73–1.35)	−0.17 (1.05–1.35)	−0.68 (0.92–1.64)	0.91 (0.6–1.4)

*Note:* The ICC indicates the degree of linear relationship between two variables. The ICC lies between −1 (indicates perfect linear negative relationship between variables) and +1 (indicates perfect linear positive relationship between variables). An ICC of 0 indicates a lack of any linear relationship. The ICC estimate is based on the 95% confidence interval (95% CI); *n* = 5. ExpHGS+ = two veterinarians with experience in HGS, ExpHGS‐ = two veterinarians without experience in HGS, non expHGS‐ = two final year veterinary students without experience in HGS.

## 4 Discussion

This study evaluates the pain expressed by horses with TMHS at rest between the painful headshaking attacks and, moreover, compares the extent of pain to horses without pain (healthy horses) and with moderate pain represented by horses with moderate gastrointestinal diseases (colic horses). As there are no cut‐off values for the HGS, we included the colic horses, as there is broad consensus that these horses are in pain and there is a need to intervene to re‐establish animal welfare. By using the HGS, we were able to show that TMHS horses experience pain in a comparable dimension to horses with moderate colic. Because no cut‐off values for the HGS have been established, the extent of pain in colic horses provides a reference for the pain category of TMHS horses. The comparison underlines that horses with severe TMHS show relevant pain conditions at rest, which are often not recognized or not regarded as a condition that needs intervention, although the well‐being of these horses is compromised.

Horses with head shaking are often of limited use to the rider due to the inability to perform in the various riding disciplines, and handling and riding might become dangerous due to involuntary head movements. For this reason, many of the affected horses are retired and spend several years in the paddock or on pasture. It is of utmost importance that these horses can still lead a life worth living. To achieve this, the head shaking triggers must be largely avoided, and not only head shaking signs, but also the pain level between episodes should be assessed regularly. This study clearly showed increased HGS pain scores in horses with severe TMHS when they were evaluated in the absence of trigger factors. There was a statistically significant increase when compared to healthy horses, with values nearly as high as in horses with gastrointestinal pain. Horses suffer silently because this species has no sounds of pain [21]. Therefore, profound evaluation of the pain signs is essential even in the absence of obvious head shaking attacks. According to the results of the study, the HGS can be a helpful tool for evaluating pain, allowing even less experienced examiners to make a good basic assessment.

In human medicine, there has been controversy regarding concomitant persistent pain in patients with TN [22]. Therefore, classification of TN into subgroups (with and without persistent pain) has been performed. About half of the human patients with TN experience concomitant persistent pain “in between the paroxysmal attacks” according to studies [22, 23, 24, 25]. Patients describe this persistent interval pain as deep, toothache‐like, aching, nagging, or dull with a median intensity of 4.6 out of 10 in a pain scale from 1 to 10 [22]. For this reason, we tried to include horses subjectively experiencing moderate pain due to gastrointestinal conditions in this study and compared pain signs in their faces to TMHS horses. It turned out that HGS scores in TMHS horses were nearly as high as in gastrointestinal conditions (median HGS of 5.6 and 6.6, respectively) and these findings support the thesis that TMHS horses also experience moderate pain between attacks.

In horses, assessing the degree of pain or evaluating stress is not easy, and the perception of whether and to what extent a horse is in pain or experiencing stress varies between owners and veterinarians [26]. Heart rate and respiratory rate could be affected by pain but are nonspecific for the presence and severity of pain [27]. Blood parameters such as cortisol, ß‐endorphins, and catecholamines could reflect stress responses that are not pain related [28, 29]. Research has found that behavioral traits are the most reliable sign when assessing pain in horses [30]. For this reason, various variables and pain scales have been developed to improve the objectivity and comparability of pain or stress reactions in horses. There are several challenges with horses. In addition to the lack of differentiation between pain and stress, the perception and expression of pain vary among individuals. Furthermore, horses express pain in completely different ways than, for example, humans or other domestic animals, such as dogs, in which voicings of pain are more common [31, 32, 33]. Subtle changes in facial expressions, such as tension in the mouth and chin, are sometimes the only expressions of pain in a species that completely lacks pain sounds [21]. Based on this fact, the so‐called pain face in horses and other animal species has been analyzed and evaluated in several studies [12, 13, 19, 34], so that the most precise features of the facial expression can contribute to the objectification of pain [12, 35]. Nevertheless, it is important to have a sound knowledge of the behavior and physiology of horses [31, 36], as a study on the HGS showed that people with no horse experience were unable to correctly analyze pain using the scale even after 30 min of training [37]. By frequently using different evaluation methods in various clinical situations, equine veterinarians and owners can improve pain recognition skills. Furthermore, the objective scales can help to evaluate and understand the facial signs in a comparable way [26, 31]. In our study, students with horse experience but who never used the HGS before were also asked to grade the horses after familiarizing themselves with the HGS for 30 min. It turned out that pain was overestimated in the healthy horses' group and underestimated in the TMHS horses, whereas pain was reliably recognized by this observer group in the gastrointestinal cases. The reason for the different scoring could not be determined in this small group of horses because there was no statistical significance between the individual FAU scores in colic and TMHS horses. Intraobserver reliability did not correlate well in the mouth‐associated variables (FAU 4 and 5) in less experienced observers. But in total, inexperienced observers with a certain “horse knowledge” and some training were able to assess painful conditions in TMHS.

One limitation of the study was that TMHS horses were only videorecorded on 2 days, so intermittent pain could have been missed. In human medicine, concomitant persistent background pain with TN was either present the whole day or at least half a day in affected patients [22]. If this is also true for the horse, it should have been discovered with the given study design. Another weakness of the study was the limited number of observers per group, as there were only two examiners per experience level. To rule out bias, intraobserver reliability was tested in this study by reevaluating five horses that had previously been scored by all examiners. Fortunately, the intraobserver reliability was good to excellent for most FAU.

Another limitation is the fact that the exact level of pain in the gastrointestinal horses was unknown, as these were subjectively chosen. Categorization by the observer subgroups nonetheless showed that the horses somewhat had a moderate level of pain. In human medicine, pain grading (on a scale of 1–10) is also subjective, and pain always has individual emotional components.

In conclusion, this study supports the fact that moderate pain is present between headshaking attacks in severely affected TMHS horses, and that quality of life needs to be regularly assessed very thoroughly and on an individual basis in these horses.

## Disclosure

The authors have nothing to report.

## Ethics Statement

The authors have nothing to report.

## Conflicts of Interest

The authors declare no conflicts of interest.
