# Erratum for “Pain Assessment of Horses With Trigeminal‐Mediated Headshaking (TMHS) at Rest Between Episodes”

**DOI:** 10.1111/jvim.70104

**Published:** 2025-04-29

1

V. Franzen , D. Reisbeck , Y. Leibl , A. Schoster , A. May ., "Pain Assessment of Horses With Trigeminal‐Mediated Headshaking (TMHS) at Rest Between Episodes," Journal of Veterinary Internal Medicine 39 (2025):e70064. doi: 10.1111/jvim.70064.40168040 PMC11960477

In the above mentioned article, Figure 1 should have used a box and whisker plot, as requested by a reviewer, with a line indicating the 50th quantile (median) of the variable of interest. The lower and upper bound of the boxes represent the 25th and 75th quantiles of the variable of interest, respectively. The lower and upper vertical lines extending from the boxes represent the lower and upper bounds of the 95% confidence interval around the distribution.

The corrected Figure 1 is shown below.

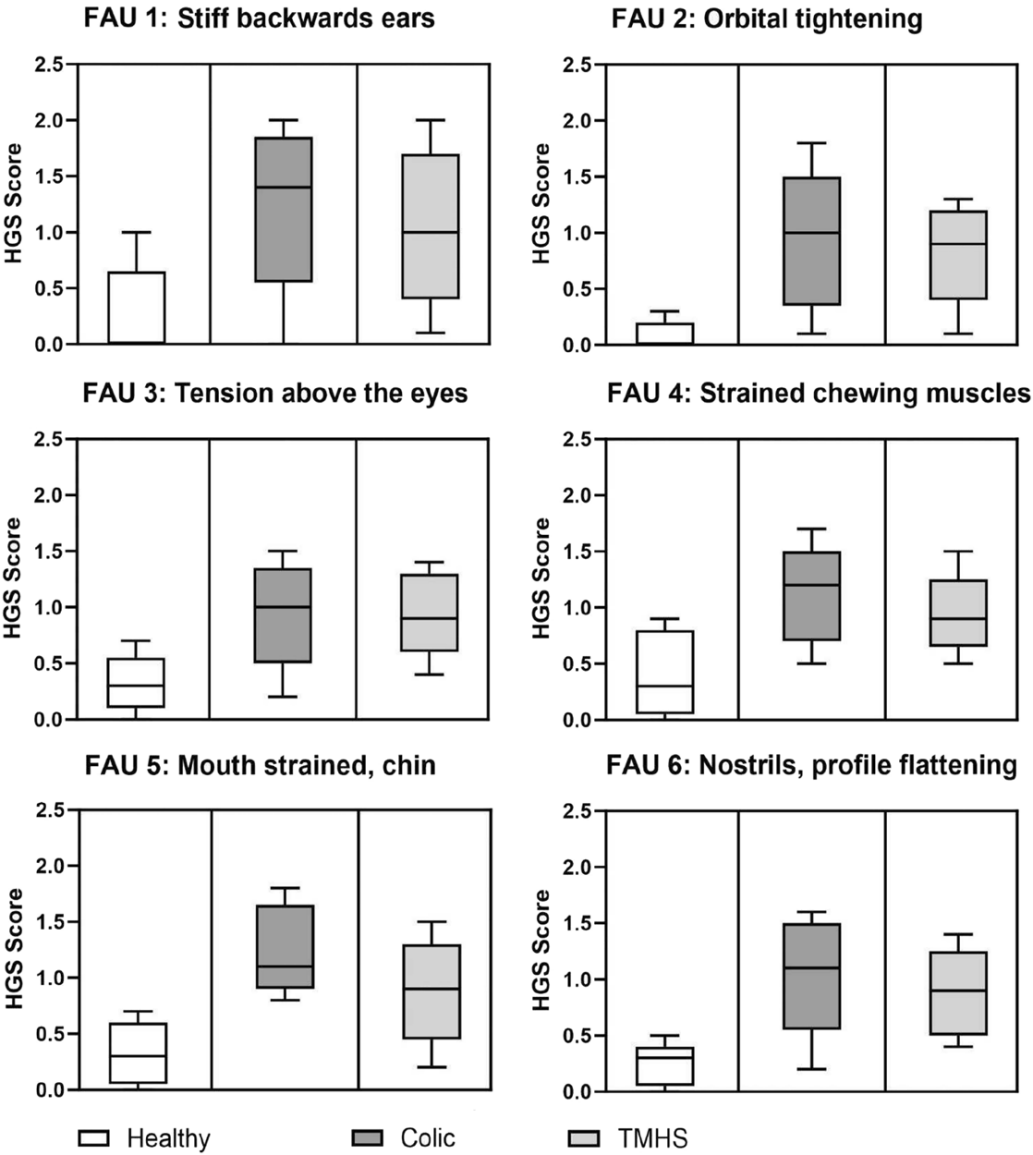

We apologize for this error.

